# Editorial: Environmental rhythms at the host-microbe interface

**DOI:** 10.3389/fgene.2023.1290552

**Published:** 2023-09-21

**Authors:** John F. Brooks, Elizabeth Heath-Heckman, Zheng Kuang

**Affiliations:** ^1^ Department of Molecular Biology, Princeton University, Princeton, NJ, United States; ^2^ Department of Integrative Biology and Department of Microbiology and Molecular Genetics, Michigan State University, East Lansing, MI, United States; ^3^ Department of Biological Sciences, Carnegie Mellon University, Pittsburgh, PA, United States

**Keywords:** circadian, circannual, microbiome, immunity, physiology

Animals form intimate associations with microorganisms, communally known as their microbiome. Much work has been performed to understand the specific microorganisms that comprise an animals’ microbiome and further how these microorganisms confer benefits to their animal hosts. Spanning from one to hundreds of microbial species, these microbiomes actively promote the health of their animal host by educating immune systems, optimizing nutrient acquisition, and blocking colonization of harmful microorganisms. Strikingly, we have come to know that the community composition and function of microbiomes oscillate across both the 24-h day-night cycle and seasons. These oscillations are tuned to the circadian and circannual rhythms of the animal and integrate microbial and animal physiology to daily and seasonal changes within the environment. In this Research Topic, we explore how biological rhythms impact microbiome community composition and function, and further discern how these changes influence host physiology and gene expression programs.

The rotation of the Earth around its axis results in an approximate 24-h day-night cycle. These rhythms in environmental light drive daily rhythms in behaviors that include feed-fasting cycles and sleep-wake cycles. Interestingly, microbiomes can coordinate biological rhythms of the animal host with the day-night cycle. This is beautifully illustrated in Ma et al., in which the microbiome coordinates an epigenetic program with the biological rhythms of the animal host. Mechanistically, microbial cues drive the rhythmic activity of histone deacetylase 3 (HDAC3) and coordinates the temporal expression of over 6,000 genes. These gene expression programs govern varied cellular processes that include ribosome biogenesis, cholesterol biosynthesis, and aerobic respiration. Further, in the absence of a microbiome, these rhythms are attenuated, exemplifying the role the microbiome plays in this coordination.

Communication between an animal host and its microbiome is bi-directional. Strikingly, members of the microbiome can tune their physiology to the biological rhythms of their animal host, indicating mechanisms by which microbial and animal physiology are synchronized. This is exemplified in Graniczkowska et al., in which the human gut commensal, *Klebsiella areogenes*, exhibits diurnal rhythms in bacterial metabolism and growth kinetics that may tune to the rhythmic production of melatonin by the animal host. Mechanistically, *K aerogenes* encodes a temperature regulated clock mechanism that coordinates gene expression to daily rhythms in the body temperature of the animal. In addition to temperature, elements of the rhythmic gene expression programs in *K. aerogenes*, was impacted by melatonin. A regulator of sleep-cycles in the animal host, melatonin is produced both in the brain and the gut. These data indicate that rhythmic melatonin production in the animal host may be another mechanism by which rhythms within the transcriptome of the microbiome are entrained.

The rotation of the Earth around the sun results in a predictive change in seasonal temperatures across geographical locations. As a result of these seasonal changes in temperature, many animals exhibit circannual behaviors that include hibernation, migration, and mating. To date, it is unclear the extent to which the microbiome impacts or responds to these behaviors. In Grond et al., diet is examined on microbiome community composition and function pre- and post-hibernation. Artic ground squirrels hibernate as long as 9 months out of the year, and during this time these animals exist under fasting conditions. Through diet manipulation prior to hibernation, Grond et al., discovered significant differences among the composition of the microbiome pre-and post-hibernation that correlated with functional activity. Interestingly, microbial activity differed more across the length of the gastrointestinal tract than under different dietary conditions. These data highlight a more significant role for host environment over diet under extreme conditions.

Both vertebrate and invertebrate animals exhibit hibernating behaviors across seasons. To understand the impact of seasonal variation and hibernation on the microbiome of invertebrate animals, Tong et al., characterized the gut microbiome of two frog species, *Rana amurensis* and *Rana dybowskii*. Overall, the microbial communities among both frog species exhibited significant variation between the summer and winter months. These seasonal changes within the microbial community correlated with changes within the functional output of the microbiome. Glycan biosynthesis was upregulated within the microbiome during hibernation and indicate a potential role for the microbiome in maintaining the metabolic requirements of the animal host during hibernation.

The mechanisms by which microbiomes influence animal health and fitness, both vertebrate and invertebrate are numerous. Moreover, we are coming to understand that communication between animals and their microbiomes are driven by a diurnal molecular dialogue. To discern the breadth of this dynamic crosstalk, we must continue to characterize the microbiomes of animal species across the world and optimize our techniques for assessment. To this end, Ma et al., characterize the microbiome of *Arborophila rufipectus*, an endangered bird species endemic to China, and Marchukov et al., compare multiple commercial kits routinely used for enrichment of microbial DNA and subsequent 16S sequencing analysis.

An animal’s microbiome confers a variety of health and fitness benefits. The advent of high-throughput sequencing technologies allowed for the examination of these microbial communities that are often refractory to cultivation. This opened the door for correlation of microbiome composition with pathology. We are now beginning to understand that these microbial communities are not static but highly dynamic, exhibiting both rhythmic oscillations across the day and seasons in accordance with the biological rhythms of the host. This discovery has revealed sampling of a single timepoint is not sufficient to devolve the complexities by which microbiomes impact animal physiology. Understanding this synchronization of host and symbiotic partners that extend across the animal kingdom both invertebrate and vertebrate, is the next frontier in host-microbiome study.

